# Biosurveillance and the Opioid Crisis in Emergency Medicine: Qualitative Study of Physician Perspectives

**DOI:** 10.2196/82865

**Published:** 2026-02-23

**Authors:** Julie A W Stilley, Mark Benton, Ashley E Douglas, Julie M Kapp

**Affiliations:** 1 Department of Emergency Medicine, University of Missouri School of Medicine, Columbia, MO, United States; 2 Department of Public Health, University of Missouri Center for Health Policy, Columbia, MO, United States; 3 Department of Public Health, University of Missouri College of Health Sciences, 731 Lewis Hall, Columbia, MO, 65201, United States, 1 5738843684

**Keywords:** emergency medicine, emergency department, qualitative, opioids, substance use disorder

## Abstract

**Background:**

In 2017, the US Department of Health and Human Services declared a national opioid crisis. In 2022, an estimated 81,806 overdose deaths involved an opioid. Emergency departments are critical in the pathway of care for providing resources and linkages to services. Studies investigating emergency medicine (EM) physicians’ perspectives on the opioid crisis have largely focused on prescribing.

**Objectives:**

To investigate EM physicians’ perspectives on response strategies to the opioid crisis of biosurveillance and linkages to care. A secondary objective was to map reported challenges and recommendations using a sequential intercept model as an actionable framework.

**Methods:**

This is a qualitative study. Six EM physicians in an academic health care system were interviewed through semi-structured interviews. Interviews were transcribed, then thematically coded and analyzed to identify cross-sector settings and barriers to care with corresponding recommendations. Settings and recommendations were mapped as a sequential intercept model.

**Results:**

EM physicians identified 9 key settings as crucial touch points in the opioid crisis: home setting, emergency medical services, patient care, clinically relevant data and information, prescriptions and pain management, predischarge coordination, outpatient resources, biosurveillance sample collection, and the external partner and administrative environment. Biosurveillance challenges included concerns about collecting biological materials for state and regional monitoring, as well as the time burden for sample collection. Linkage to care challenges included social determinants of health and limited outpatient care access. Recommendations were specific to each setting and included care coordination and fostering cross-sector partnerships. A patient-centered approach and better integration of community resources were emphasized.

**Conclusions:**

The service delivery culture is of acute and episodic care but needs to more seamlessly address care across its fragmented multicomponent complex system. EM physicians face systemic challenges and provide actionable recommendations to promote comprehensive care to patients presenting to the emergency department with opioid-related complaints.

## Introduction

In 2017, the US Department of Health and Human Services declared a national opioid crisis [1]. In 2025, the public health emergency declaration was renewed [2]. This crisis has evolved from overdose deaths related primarily to prescription painkillers, to heroin, to illicitly manufactured fentanyl and other synthetic opioids [3]. Recently, opioid overdose deaths often involve polysubstance use [4]. In 2022, an estimated 81,806 overdose deaths involved an opioid [5], and 11.8% of adults reported a past substance use problem, with over 70% identifying as in recovery [6]. Despite recent declining trends in overdose mortality, fentanyl-involved nonfatal overdose emergency department (ED) visits remain high [7].

The federal public health response [8] to the opioid crisis recommended key public health and surveillance components: strengthening public health data reporting and collection to improve the timeliness and specificity of data to inform a real-time public health response; and improving access to prevention, treatment, and recovery support services to prevent the health, social, and economic consequences associated with opioid addiction while enabling individuals to achieve long-term recovery. Federal response has included using ED and emergency medical services (EMS) data to track and analyze morbidity trends, and building systems to facilitate better linkages to treatment, such as emergency room peer patient navigators. Central to these strategies is establishing partnerships among public health, public safety, harm reduction, and clinical and health settings.

Public health surveillance has been actualized through prescription drug monitoring programs (PDMPs) to minimize high-risk opioid prescribing. In 2022, 21 states reported 96,332 nonfatal opioid-related ED visits [9]. ED patients frequently present with chief complaints of pain, requiring emergency medicine (EM) providers to balance effective pain management with the risks associated with prescription opioids [10]. EDs are critical touchpoints for providing persons who use drugs with overdose prevention resources and linkages to care, as those who experience a nonfatal opioid overdose are at increased risk of a subsequent overdose [11].

Previous research involving EM physicians has largely focused on prescribing. ED providers expressed helplessness, sadness, and frustration toward current approaches to treating patients with opioid use disorder (OUD) [12,13]. Many felt unprepared to discuss buprenorphine (an evidence-based treatment option) with patients [12]. Providers expressed clinician- and system-level barriers to initiating buprenorphine, with recommendations for improved training opportunities, clinical protocols, and referral systems for outpatient follow-up [12]. Providers generally support opioid prescribing guidelines for EDs [14,15]; however, they described a need for increased administrative support, improved PDMP efficiency, and increased patient involvement in the opioid prescribing process [15].

Biosurveillance is the process of gathering, integrating, interpreting, and communicating essential information related to all-hazards threats or disease activity affecting human, animal, or plant health to achieve early detection and warning, contribute to overall situational awareness of the health aspects of an incident, and enable better decision-making at all levels [16]. In the context of the opioid crisis, this might include toxicology testing of drug products or individuals’ biological samples (such as blood or urine) to track and monitor drug trends and overdose outbreaks [17]. Individuals admitted to the ED for an overdose would be potential candidates for testing.

While PDMPs and prescribing guidelines have been studied as response strategies to the opioid crisis, little is known about EM physicians’ perspectives on biosurveillance and linkages to care, especially across the care continuum. This qualitative study analyzes data from semistructured interviews with EM physicians. Our primary objective is to investigate EM physicians’ perspectives on response strategies to the opioid crisis related to biosurveillance and linkages to care. Our secondary objective is to map their reported challenges and recommendations in a sequential intercept model (SIM) as an actionable framework. SIM mapping of EM physicians’ perspectives in a cross-sector model is innovative, given that historically this modeling has been used to inform community-based responses to the engagement of people with mental health conditions and substance use disorders (SUD) in the criminal justice system [18-20].

## Methods

### Study Design

This study is a qualitative investigation of EM physicians’ perspectives on biosurveillance and linkage-to-care responses to the opioid crisis, including barriers and recommendations along the patient care continuum. A content analysis of semistructured interviews was applied to a purposive sample of EM physicians with clinical responsibilities practicing in the ED.

### Setting

The setting was an academic tertiary EM department in a Midwestern community in a county of about 200,000 residents. This setting provides the only Level I trauma center and the only board-certified team of EM physicians in the region. The department has a catchment area of 30 mostly rural counties spanning 190 miles in length and 125 miles in width.

### Participants

The project was announced to EM physicians during a faculty meeting, with interested participants recruited through in-person invitations. Recruitment targeted diversity in physician roles. Personal invitations included a brief summary stating that the study aimed to understand physicians’ experiences in the ED, outlining that participation would involve a 30-minute semistructured interview, and emphasizing the value of their specific professional perspective. All six board-certified EM physicians invited for interviews agreed to participate. The participants were previously familiar with the interviewer (author JAWS). In addition to clinical duties, each participant maintained at least one of the following administrative foci or subspecialty board certifications: medical administration; medical student or EM resident education; EMS; pediatric EM; or academic research specializing in OUD. All participants were trained at outside residency and fellowship programs across the country, and the majority practiced as attending physicians outside of Missouri prior to their current role.

### Data Collection and Interview Process

A first draft of interview questions was developed in April 2023 based on EM physician experiences in their roles, initiatives to enhance biosurveillance (eg, the Centers for Disease Control and Prevention’s Enhanced State Opioid Overdose Surveillance program [8]), and their perspectives on care coordination across a broad cross-sector. Two authors (JAWS and JMK) collaborated on refining the questions with an EM physician sample in mind. Textbox 1 shows the final interview questions. Semistructured interviews occurred between July and August 2023 without incentives. One interviewer familiar with the participants (JAWS) conducted the interviews. Participants 1 and 2 volunteered to participate together given their availability; the remaining participants opted for one-on-one interviews. Participants received questions on the day of their interview. Interviews occurred in a private office. The interviewer read each question aloud, and follow-up questions were asked as the interview progressed. Interviews were completed in approximately 30 minutes (mean 32, range 25‐47 min). In the case of the dual interview, each participant answered the questions individually and then was allowed to expand upon the other’s responses. To confirm that the dual interviewees did not influence one another, each was independently asked later if they were satisfied with their answers, and both indicated in the affirmative. All interviews were audio recorded with permission.

Textbox 1. **Focus group interview questions.**

1. How is the opioid crisis affecting your job in the emergency department (ED)?

- Prompt with “What challenges are you facing in your day to day?”
- Prompt with “How has this evolved as the opioid crisis evolved, for example, inclusion of fentanyl, xylazine, etc.?”
- Prompt with “What solutions have you thought might work?”

2. How do you think social determinants of health affect what you see in the ED in regards to opioids?

- Prompt with “What resources are hard to get? For your practice and for specific patients?”
- Prompt with “How do you identify any gaps in care? How has the ED tried to provide wrap around services or tried to make community connections?”

3. There is interest from the state in collecting samples from overdose patients and sending them to a state laboratory for testing. Do you think our hospital would be interested in participating?

- Prompt with “Why do you think our hospital wouldn’t want to?”
- Prompt with “What if it was only up to the ED staff to send in samples?”
- If there is robust conversation about this question, provide more context, “The state is hoping to collaborate with MHA (Missouri Hospital Association) to assist in this effort. Do you have any thoughts about that?”

4. How do you currently learn information or current data about the state of the opioid crisis? Locally? Statewide? Nationally?

- Prompt with “How would you like to learn about it?”
- Prompt with “How would you use the information?”
- Prompt with “How can we be a good partner to you when getting you the most up to date information?”

5. How can we best connect with first responders, correctional systems, and others?

6. What’s your experience with EPICC (Engaging Patients in Care Coordination) in the ED? Is their presence helpful to patients at risk for overdose?

7. Do you know of any other acute resources that your patients can use outside of EPICC?

### Data Analysis

Audio recordings of the interviews were transcribed by two authors using slowed playback. Transcripts were organized into a Microsoft Excel spreadsheet, segmented into natural conversational breakpoints by row, resulting in 191 rows of data referred to as “observations.” Authors identified predominant patterns and themes through content analysis. To reduce bias, authors involved in developing initial codes and applying those codes were not the same as the author who collected the data. First, two authors (MB and JMK) independently reviewed organized transcripts to inductively develop two preliminary sets of codes. Authors’ goals for interpreting the data included identifying physician perspectives related to actionable opioid response strategies and explanations of patient care touchpoints along the treatment continuum. Next, all authors discussed and reviewed these codes, drawing on their areas of expertise to create a revised coding scheme. Two authors (MB and AED) then independently applied this scheme to 10% of observations. All authors discussed the results of the 10% of coded observations to better understand how interpretation was applied and to continue the refinement of codes until final. This was accomplished with researchers tracking coding spreadsheets as they developed, discussing disagreements in person and via email to clarify coding discrepancies, and debriefing on the coding process until final agreement was reached. Two authors (MB and AED) then independently coded remaining observations for the two categories described below using final codes.

The first category of coding was the “setting,” which included the context of intervention points for a person presenting with suspected overdose or suspected OUD (see Table 1). Settings were categorized into 9 areas: home setting, EMS, ED (including patient care, prescriptions/pain management, predischarge coordination, and clinically relevant data), outpatient resources, biosurveillance sample collection, and the external partner/administrative environment. Settings were grouped as 3 phases: *prehospital* (prior to entry to the ED), *ED* (the span of medical care activities in that setting), and *cross-sector partnerships* (administrative or organizational setting that includes structure, partnerships, resources, and regulations). The second category of coding was challenges and barriers (C/B) and actionable “recommendations” for intervening in those settings.

**Table 1.** Codes and definitions.

Setting code	Definition
Home setting	Outside of the health care system, social conditions, housing, food insecurity, transportation, access to care, telehealth, geography.
Emergency medical services	Exclusively in the care of EMS^a^, regardless of the location of EMS activities.
Patient care	Patients in the care of ED^b^ personnel, the process of clinical treatment, clinical decision-making, Narcan-revivals, trends in diagnoses.
Clinically relevant data and information	How EM^c^ providers prefer to receive information, timeline of information, geography of data disseminated, information sources, information processes.
Prescriptions/pain management	Considerations of substance use disorder potential while prescribing.
Predischarge coordination	Peer counseling, peer support, peer navigation, peer education, coaching, social work, coordinating resources, warm handoff.
Outpatient resources	Long-term medication-assisted therapy, rehabilitation, at-home Narcan resources, mental health care, discharge by EMS at the site of emergency.
Biosurveillance sample collection	Sample collection, sample testing/analysis, patient confidentiality in testing, resources needed for sampling, workflow related to samples.
External partner or administrative environment	Hospital coordinating bodies, interactions with law enforcement/justice systems/courts, rules and policies, partnership with state government.

a EMS: emergency medical services.

b ED: emergency department.

c EM: emergency medicine.

For confirmability of coding, Stata version 16.1 was used to assess agreement between coders. For setting, 2 authors (MB and AED) agreed on 73% of codes (κ=0.6874, substantial agreement [21]), and for C/B or recommendations, 2 authors agreed on 83% of codes (κ=0.6407, substantial agreement). A third and a fourth author separately interpreted remaining disagreements independent of prior coding, resulting in each observation having one setting and one C/B or recommendation categorization.

Observations were not weighted, with each observation carrying the same importance during analysis. The frequency of codes was tabulated to approximate how commonly a topic was discussed, suggesting participants’ emphasis on that topic.

Example quotes were selected for each C/B and recommendation for each setting. Quotes were lightly edited for readability (eg, spelling corrections or removal of disfluencies) but retained their original meaning. Ellipses indicate removed text, and relevant unspoken contextual details are added in brackets.

Identified settings informed the creation of a SIM map to visually summarize the sequential span of potential intervention points and recommendations for those settings. Categorization of settings into phases was developed by all authors inductively, based on pattern emergence and interpretation from author expertise areas (ie, EM, epidemiology, behavioral neuroscience and addiction science, public policy, and program evaluation), with the goal of providing a visual representation of a potential sequence of settings and intervention points from the patient’s perspective after overdose. Bullet points were added under each setting to reflect key recommendations provided by physicians. SIM mapping has previously been used to display how people move through the criminal justice system with the intention of generating considerations for interception points [18,19].

### Ethical Considerations

This study was reviewed and approved by the University of Missouri Institutional Review Board (IRB 2101376). This study is a secondary analysis of previously collected, deidentified transcripts from a program evaluation and therefore did not require additional consent.

## Results

### Descriptive Characteristics

Table 2 reports descriptive information for coded observations. The most common setting mentioned was clinical decision support, with 46 out of 191 (24%) observations coded. The least common settings were prescriptions or pain management and EMS, each with only 4 out of 191 (2%) observations coded. Observations included more C/Bs (122/191, 64%) than recommendations for improvement (69/191, 36%). The phase with the most observations was the ED (110/191, 58%), followed by cross-sector partnerships (58/191, 30%) and preclinical (23/191, 12%). The percentages of recommendations within phases included 13% (3/23) for prehospital, 40% (44/110) for ED, and 38% (22/58) for cross-sector partnerships.

**Table 2.** Distribution of settings, challenge/barrier, and recommendations.

Phase and setting	Setting, n/N (%)	C/B^a^ and recommendations, n/N (%)
Prehospital (n=23)		
Home setting	19/191 (10%)	C/B: 16/19 (84%) Recommendations: 3/19 (16%)
Emergency medical services	4/191 (2%)	C/B: 4/4 (100%) Recommendations: 0/4 (0%)
Emergency department (n=110)		
Patient care	27/191 (14%)	C/B: 21/27 (78%) Recommendations: 6/27 (22%)
Clinically relevant data and information	46/191 (24%)	C/B: 24/46 (52%) Recommendations: 22/46 (48%)
Prescriptions/pain management	4/191 (2%)	C/B: 4/4 (100%) Recommendations: 0/4 (0%)
Predischarge coordination	33/191 (17%)	C/B: 17/33 (52%) Recommendations: 16/33 (48%)
Cross-sector partnerships (n=58)		
Outpatient resources	10/191 (5%)	C/B: 6/10 (60%) Recommendations: 4/10 (40%)
Biosurveillance sample collection	21/191 (11%)	C/B: 14/21 (67%) Recommendations: 7/21 (33%)
External partner/administrative environment	27/191 (14%)	C/B: 16/27 (59%) Recommendations: 11/27 (41%)
Total	191/191 (100%)	C/B: 122/191 (64%) Recommendations: 69/191 (36%)

a C/B: challenge/barrier.

Table 3 reports examples of C/Bs and recommendations in each setting. The leftmost column shows each setting, the center column shows quotes related to C/Bs, and the rightmost column shows quotes related to recommendations.

**Table 3.** Demonstrative C/B^a^ and recommendations from each setting.

Setting	ID: Example C/B	ID: Example recommendation
Home setting	1: From a standpoint of rural agencies, there's a lot less resources the farther out you go…There are not as many treatment centers…and a lot of those people…cannot drive 30 [minutes to] an hour to go through outpatient treatment.	4: It has to be a free service. A phone number that they can call and talk to someone…Everyone these days needs therapy, so probably some sort of free therapy.
EMS	2: I think for EMS^b^, a lot of these substance abuse patients also have dual diagnoses with psychiatric complaints. So, there can be a lot of overlap and trying to figure out the primary problem for that day and sometimes those [behavioral] issues can lead to safety concerns.	Not applicable
Patient care	6: I have had to call codes [as deceased and declared deceased] on people who are opiate overdosed. I've gotten people back [from death] from opioid overdose and seen their reaction. It ranges from sorrow and dismay…to annoyed and angry at me that I have taken away their high. 5: I think that fentanyl has been an increased issue across the board.	3: I ask about whether they would be interested in resources for substance use treatment and whether they have a primary care doctor who they feel comfortable talking with about this subject. 4: Usually it's asking a pretty thorough history and including those additional historical questions, like social history and past medical history. 6: I do my very best to attempt to be an ally... I always thank people for their honesty, particularly when they tell me something shameful or taboo or whatever…it’s my perception that it helps build rapport, and lets them know that my main goal is to help them from a medical perspective.
Clinically relevant data and information	3: But no matter which opioid you're using, if you come in overdosed to my department, I'm getting you a Narcan. 4: I’ve been spammed by a whole bunch of medical email listservs that I'm somehow on now, and there's always something about [the state of the opioid crisis], but it never seems like it's related to my population. 4: I do not feel like it’s timely and I feel like I almost don’t get any information. 5: I don’t know a whole lot about what’s going on in the state or the county.	1: If a dashboard were built, even a 30-day delay would be too much to be actionable. 2: [It would be helpful for the dashboard to show] where the event occurred, where the 911 call or where the hospital was. County level is plenty…We don’t need any information about the patient themselves. 2: I really need new data, real time information. Last year's data is interesting to know, but it’s not actionable. 6: If I'm on an email list, the odds of me actively looking at it are probably a lot lower…Honestly, I'd probably be more apt to read it if it came from our admin.
Prescriptions/pain management	6: I try to be judicious of like, how much [opioid] do I think [the patient] needs? And how much am I ok prescribing them? In the literature, it’s like less than 10 pills is all it can take to get people addicted to opioids. 6: I feel more people are reticent to get fentanyl when they come to see us, because they’ve heard about fentanyl in the media and whatever.	Not applicable
Predischarge coordination	2: If you leave a pamphlet, the likelihood of somebody calling [for treatment] is small versus if you make the call yourself and set it up. But to attempt to do that in a hospital setting, time may be an issue. 4: Our social worker has very limited hours and she is stretched thin based on what’s going on in the ED.	2: I would like to have an ED^c^ social worker that is present 24/7. 4: It would be beneficial to have a therapist in the ED. Having a social worker available 24 hours a day would be huge too. 6: It would be nice to have a social worker 24/7.
Outpatient resources	3: The other thing that is acutely available and very useful is a script for Narcan or even better, an actual dose of Narcan to take home with them from the ED. 6: I am aware that there are detox houses. Or houses where people have some other services.	1: Access to some sort of treatment center, even just a hallway or one room in a local public health [organization], would be a huge benefit for a lot of these patients. Somewhere they can go locally…where they can have a social worker help, arrange for resources once they've had some medical evaluation, and get plugged into continued outpatient resources. 3: I wish we had a detox slash treatment unit associated with our hospital.
Biosurveillance sample collection	2: There may be some difficulty in workflow, especially on busy days. 3: We are already understaffed, underfunded, and overstretched in the ED. To give us one more thing to do without providing the resources to do it is not helpful. 4: A lot of concerns for patient privacy, realistically. I think if you have more than one identifier…you can probably track that patient back.	4: [You could collect the] ZIP Code of where the overdose was. Just so they can see if there are pockets, or certain communities that have a higher incidence of overdose, and maybe law enforcement could look into that further. But even that’s a bit dicey because they are getting information that was provided by the hospital. Yikes. 5: If you’re collecting information about the person it has to be voluntary. I don't think you have the option of obtaining information, personal information of any kind, and samples, without consent. If it’s a way to collect samples so that there's no patient information, then I think that would be acceptable. 5: I think it can be done anonymously versus having to have minimum patient information on it. 6: Patient autonomy is a tenet of medicine.
External partner/administrative environment	4: I think the MHA^d^ is great because it can outreach to all the hospitals in Missouri. They do some really great things on different initiatives. So, maybe they could help with legal issues, like what are the minimum necessary requirements and how can we keep hospitals and physicians safe. 5: I don't have great insight into the system…I mean, we don't really deal with the aspect of if someone relapses. We treat them and we’re going to deal with their medical problem, and then we don't really deal with the justice side. That's not really our area to take part in. 6: As soon as I was allowed to do it, I had an X-waiver, before it became a thing you didn’t have to have anymore.	1: I think MHA has administrative capabilities. They can run a dashboard and can probably run a dashboard better than the state. 4: I see them as a higher-level organization. Getting the general counsel kind of things together and making sure all the labs are run the same…So if they could come up with like 1 clear protocol for everyone to use that would be beneficial to the whole state. 6: I think MAT^e^ in the correction[al] system would be great.

a C/B: challenge/barrier.

b EMS: emergency medical services.

c ED: emergency department.

d MHA: Missouri Hospital Association.

e MAT: medication assisted treatment.

### Home Setting

Participants often mentioned social determinants of health (SDH), which included challenges such as rural geography, income, language barriers, employment, insurance, food access, and disability status. Treatment can be especially difficult to access for people with low income or no insurance.

Many treatment programs require that you pay for them and if you don’t have a job with health insurance you are unlikely to be able to pay for it. And if you don’t have a good leave policy at your job, you risk losing your job. So, if you have the money to pay for treatment, and if you have a good job that understands taking time off for a health issue, you’re going to have a much easier time treating the problem than if you work graveyard shift at a factory or at the 7/11 and you’re living paycheck to paycheck, and you can’t afford to miss a single shift.

[Participant 3]

Participants recommended improving access to or offering free therapeutic community services, particularly through telehealth.

### Emergency Medical Services

A call to EMS is often a first step after overdose. EMS patients can have complex needs, creating barriers to identifying the root cause of a call. Participant 1 stated, “dual diagnoses definitely are frequent with SUD if it is psychiatric disorders as well.”

### Patient Care

Participants reported an increase in opioid overdoses and a shift from heroin toward fentanyl. Patients’ reactions after administration of naloxone varied from anger to dismay; some are open to discussing treatment, but Participant 4 noted some “are not even willing to have that conversation.” Participant 4 recognized the need to involve families:

[The opioid epidemic] affects their family members, loved ones, or they know someone…if a patient wants to come in for withdrawal symptoms…do we have appropriate outpatient resources for the patients? And what do we do with their families and how do we refer them on?

[Participant 4]

Recommendations included offering treatment information to patients. Additionally, Participant 4 recommended improving education for medical students on SDH screening, while Participant 3 discussed screening for the trustworthiness of the drug supplier:

Ask “where do you live? Do you have a safe place to stay? Do you have transportation?”…We need to teach residents and physicians to just keep asking those questions, even though they might have some information in front of them because you can’t always rely on what’s in the computer…Sitting down at their level, not interrupting, all those things so they feel comfortable talking to you and being nonjudgmental.

[Participant 4]

One of the things I actually do ask people when they have a bad trip, and they’re concerned that they may have been slipped something, is whether they got their [drugs] from a usual supplier that they trust, or whether it’s somebody new that they have no idea how reliable they are.

[Participant 3]

Participants suggested not assuming existing information about patients in the electronic record was correct and to be diligent in gathering information to best help patients.

### Clinically Relevant Data and Information

Participants wanted surveillance information disseminated about drug trends and opioid overdoses but were unsure that this would meaningfully alter clinical care and instead be useful for population-level outreach and intervention planning. Participant 3 stated, “no matter which opioid you’re using, if you come in overdosed I’m getting you a Narcan.”

Biosurveillance information may help if timely, relevant, and direct. Participants wanted information on local drug trends or overdoses, but it would need to be within the occurrence week to be actionable. Participant 3 reported:

If a dashboard were built, even a 30-day delay would be too much to be actionable. You’d almost have to have a weekly update.

The best way to receive information is communication from their institution. Some also suggested communicating through professional association (eg, American College of Emergency Physicians) emails.

### Prescriptions/Pain Management

Participant 6 spoke to the tension of judiciously prescribing opioids for managing patients’ pain while also avoiding addiction potential. They also spoke to the experience of working in a state with a robust PDMP versus a state [like Missouri] with a nascent PDMP:

I feel more people are reticent to get fentanyl when they come to see us, because they’ve heard about fentanyl in the media…we’re a much more controlled setting than getting your bundle off the street…fentanyl has more bad press around it and [people] don’t want to use it…Pills around here are more accessible. Where I was previously, I think heroin was more accessible. I came from a state that had a strict and robust PDMP…it was much easier [in the previous state] to look up people who had been getting prescriptions.

Only one participant offered prescription/pain management setting observations, and no one offered recommendations for that setting.

### Predischarge Coordination

Patients were perceived as less likely to receive post-ED care if left to self-coordinate than if coordination was provided. This puts a burden on ED physicians, and social work resources are at capacity. An increase in dedicated care coordination resources would be beneficial. Participant 4 noted:

There’s a lot more [treatment] resources for people to get compared to 10‐15 years ago. Because it’s more talked about, not as scary for patients to admit to it, and they’re more forthcoming… So, then we can actually get them the help that they need…[If they need] medication assisted treatment [MAT]…the resources are a lot more plentiful. I love the [Engaging Patients in Care Coordination] EPICC program because it’s like a peer coach. And I’ve had really good luck with that.

Participants need all-hours social worker positions in the ED to assist in care coordination, which is seen as the responsibility of ancillary services. Participants spoke highly of the Missouri Hospital Association (MHA) EPICC program, based on the screening, brief intervention, and referral to treatment model that links opioid overdose survivors at a point of crisis to community-based care via peer outreach across institutional and community settings. Participant 6 said:

My experience with EPICC has been that they’re an excellent resource. I appreciate that they call and/or see the person in real time.

### Outpatient Resources

Participants had limited knowledge of OUD outpatient resources in communities, such as residential treatment centers, MAT, or longer-term mental health care. Participants encouraged patients to seek and receive care and offered what their hospital had available; however, connecting to community resources for outpatient treatment was beyond their role. Participants were challenged with getting patients access to ongoing outpatient treatment. For example, Participant 5 said, “many of the places that address addiction don’t take pediatric patients.”

Primary care is so short staffed…If you come in ready to change and ready to get treatment, but it’s going to be 3 months before you can see anybody, then the chances you can maintain that on your own are pretty small.

[Participant 3]

Recommendations included improved processes to connect patients to local clinical- or community-based resources. Participant 3 stated:

Opioid withdrawal is miserable and may make you wish you were dead but will not actually kill you, so it’s very seldom that we admit somebody to the hospital for that purpose.

However, the importance of outpatient resources was noted. Participant 1 said:

Sobriety centers are [something] we see a lot of in urban areas, but most of those are funded by health systems trying to divert patients out of the ED…I think something locally that enables the patient to stay there… from the standpoint of the ED, tele-psychiatry is sorely needed.

### Biosurveillance Sample Collection

Participants expressed concern about collecting biological materials and sending them to a central location for state and regional opioid use monitoring. Challenges included the time burden, a lack of clarity on whether samples would be collected via kits or tubes, and funding. Participant 1 said:

I think workflow is going to be huge…are we using our own equipment... is the state providing a kit…a whole kit you have to put the information in, that’s a barrier.

Participants believed ED staff roles in collecting and shipping samples should be minimized. Participants had concerns about patient privacy and consent, seeing it as inappropriate to sample and test patients without consent. Participant 6 asked, “is that a patient gets-to-decide kind of question?” and stated, “it’s their body, it’s their choice.” Even with consent, participants reported concerns about potential law enforcement consequences; it was viewed as inappropriate to attach personal information to samples.

Participants suggested that a partnership with MHA could be beneficial if they provide staff to process samples. Participants strongly recommended obtaining patient consent for sampling and minimizing identifiable information on samples. Participants speculated that only the ZIP code and date might be needed.

### External Partner/Administrative Environment

Challenges included siloing between organizations. For example, physicians or other medical professionals did not often interact with the justice system. Concurrently, there was support for modernizing opioid treatment in criminal justice systems, such as providing MAT in correctional systems. Participants also spoke of challenges with administrative red tape that created barriers to clinical practice. One participant described the importance of obtaining an X-waiver from the Drug Enforcement Agency when that authorization and registration was required.

While participants appreciated EPICC and other care coordination services, Participant 2 also acknowledged the challenge of coordinating external resources:

[Care coordination and referrals is] kind of scattered with programs like EPICC, the local clinic, and some of the other disorder clinics and stuff. [It’s challenging]…having to coordinate several different programs that overlap and interact but are different.

Participants viewed MHA as capable and well-positioned to provide administrative support for dissemination of biosurveillance information to Missouri hospitals.

### Sequential Intercept Model

**Figure 1.**
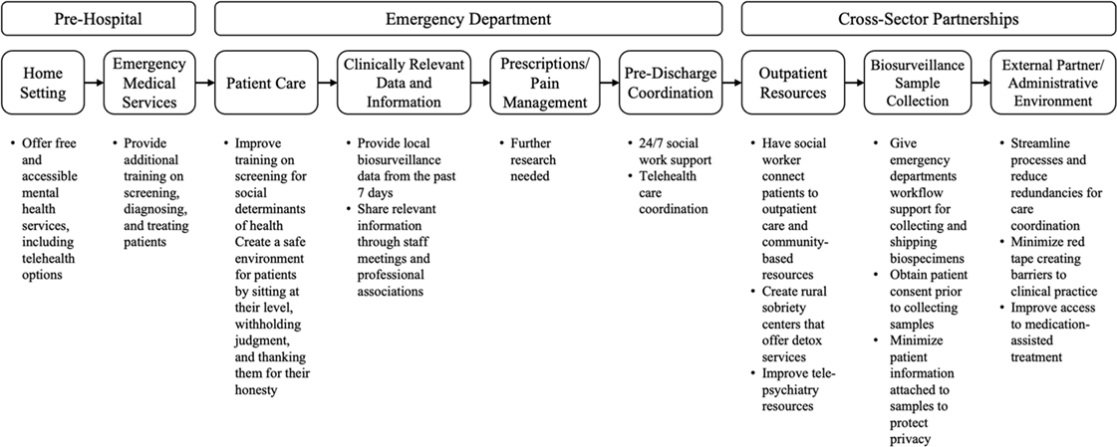
Sequential intercept model mapping to emergency department physicians’ perspectives on the opioid crisis.

Figure 1 shows a SIM map based on the findings reported above, with summary recommendations bulleted below the corresponding setting. This SIM map summarizes and integrates the coded data from EM physicians into a visual representation of a patient’s potential overdose experience across sequential settings (home through outpatient), highlighting contextual opportunities for intervention based on participant recommendations for action.

## Discussion

### Principal Findings

This is the first study to investigate EM physicians’ perspectives on actionable response strategies to the opioid crisis of biosurveillance and linkages to care throughout the care continuum, and then to map their reported challenges and recommendations in a SIM as an actionable framework across the sequence of patient settings. Key patterns across settings include a desire for actionable, practical support with clear workflows. Workflow and implementation burden are concerns across settings given limited staffing and lack of capacity. There is also significant variability in resource availability and community infrastructure, limiting local options and creating constraints.

Regarding biosurveillance dashboards, given the acute and rapid nature of EM, with physicians making complex decisions in the absence of ideal data, dashboards are not perceived as actionable unless they are current and within a local context. New knowledge of the overdose landscape from dashboards is not likely to alter the standard of care. Participants prefer that critical information be communicated through trusted sources such as professional organizations. Participants expressed concern about privacy and patients’ ability to consent to sample collection. Concerns related to patient privacy, stigma, and drug-related prosecution are common and necessitate maintaining strict confidentiality [22]. The Association of Public Health Laboratories recommends routine confidentiality training for staff, establishing data use agreements between parties involved in data sharing, using unique identifiers in place of identifiable data, and sharing aggregate data with partners [23].

Regarding linkages to care, the service delivery culture is acute and episodic care but needs to more seamlessly address care across its multicomponent complex system. Participant responses reflect fragmentation between systems (especially EMS, ED clinical care including predischarge coordination, and outpatient treatment), with weak care transitions, particularly for patients with social or behavioral health needs. Participant recommendations inform federal goals for decreasing barriers to care. Sobriety centers are needed, along with 24/7 social work support to connect patients with resources, including outpatient treatment and establishing primary care. For rural patient challenges of availability and distance to treatment, participants recommended therapy by phone or telehealth. Telehealth-delivered substance use interventions are generally shown to be effective alternatives to in-person treatment, although further research on this topic is needed [24]. Finally, participants indicated that pediatric populations are particularly challenged in accessing SUD resources.

Participant recommendations inform education and training for peers, screening patients to determine whether they have trusted primary care, and their willingness to pursue SUD resources. Key patterns mentioned across settings include stigma, judgment, and internal tension about prescribing or referring. Patient–provider interaction recommendations included creating a safe space for patients by sitting at their level, not interrupting, withholding judgment, thanking patients for their honesty, and treating patients humanely. Participants recommended updating didactics for trainees to include screening patients for SDH. Participants also acknowledged stigma and bias that other providers can hold against the OUD population and the detrimental effect this can have on learners’ perspectives. Many of these recommendations align with those reported by ED patients with OUD; patients have described experiencing stigma in the ED, indicating a desire to be treated with respect and for their autonomy to be recognized [25]. To accomplish these goals, however, requires a significant level of emotional and relational labor across care settings to build rapport, create safe environments, manage patient trust, and minimize compassion fatigue.

EMS workers are important partners in addressing the opioid crisis. More research is needed in the prehospital setting to characterize the challenges of EMS providers. EMS providers would benefit from expanded clinical training to effectively respond to psychiatric calls and drug overdoses [26]. This is consistent with EM participants’ perspectives on challenges with psychiatric conditions of the patients, dual diagnoses, and the safety of the EMS crew. Federal initiatives acknowledge the role of EMS, but more research is needed on their lived experience in responding to the opioid crisis.

### Strengths and Limitations

Although our findings may be limited by state-specific laws, a small sample size, and the local, single-site setting, there was surprising consistency between the views of our participants and those in another study, enhancing trustworthiness and suggesting transferability of findings to similar contexts. Specifically, findings are consistent with those from a study conducted in New Hampshire, which recommended naloxone prescriptions to those presenting with an overdose, 24-hour on-call recovery coaches, providing resources, and supporting needle syringe programs and prevention programs to address pain [13]. Still, given the limited sample size, findings should be considered exploratory. Selection bias was not present in this study, as all participants who were approached agreed to participate. Confirmability of coding was mitigated given the author conducting interviews did not also code the data and was tested with Kappa statistics. Findings represent an in-depth analysis of EM physicians’ lived experiences, although the sample size was small and limited to a single site, participants’ experience working in previous settings enriched the breadth of their knowledge and perspectives.

### Conclusions

Prior studies have examined barriers within a single clinical environment and focused on prescribing; this study provides a rare cross-setting analysis of EM physicians’ perspectives on the opioid crisis, including the home setting, EMS, ED (including pain management and predischarge coordination), outpatient resources, and external partnerships. This study provides an opportunity to hear directly from EM physicians about the challenges they face as frontline workers to the opioid crisis and offers actionable recommendations. Findings reveal system-level constraints, emotional and cognitive burdens, stigma, judgment, and the need for additional training and resources for the providers themselves. Organizing findings as a SIM map summarizes and disseminates information into a useful visual to facilitate actionable discussion with stakeholders about the end-to-end care coordination pathway and the barriers to recovery.

## Abbreviations

ED: emergency department
EM: emergency medicine
EMS: emergency medical services
EPICC: Engaging Patients in Care Coordination
MAT: medication-assisted treatment
MHA: Missouri Hospital Association
OUD: opioid use disorder
PDMP: prescription drug monitoring program
SDH: social determinants of health
SIM: sequential intercept model
SUD: substance use disorder
